# Videothoracoscopy in Pleural Empyema Following Methicillin-Resistant *Staphylococcus aureus* (MRSA) Lung Infection

**DOI:** 10.1100/tsw.2009.91

**Published:** 2009-08-01

**Authors:** Duilio Divisi, Giovanna Imbriglio, Roberto Crisci

**Affiliations:** Department of Thoracic Surgery, University of l'Aquila, ”G. Mazzini“ Hospital, Teramo, Italy

**Keywords:** pleural empyema, MRSA lung infection, videothoracoscopy, surgical treatment

## Abstract

Our study shows the different therapeutic procedures in 64 patients with pleural effusion due to MRSA pneumonia. The thoracostomy tube associated with pleural washing was decisive in 10 simple effusion patients. Video-assisted thoracic surgery allowed a complete resolution of the disease in 22 complex parapneumonic effusion patients. In 20 of 32 patients with frank pus in the pleural cavity, the videothoracoscopic insufflation of carbon dioxide (CO_2_) before thoracotomy facilitated the dissection of the lung tissue. In 12 patients, this approach was not applied because of cardiac insufficiency. Videothoracoscopy and decortication after thoracotomy ensured the recovery of functions.

## INTRODUCTION

The increased incidence of nosocomial diseases, of acquired immunodeficiencies (drug users, incarcerated or homeless people, neoplastic patients treated with chemotherapy), and of an improper exposure to antibiotics explains the real increment of methicillin-resistant *Staphylococcus aureus* (MRSA) pleural empyema. Beam et al.[1] considered the two most important risk factors: (1) recent hospitalization and (2) chronic illness needing reiterated home care. The histological evaluation allows the differential diagnosis of the benign epithelial inclusion, characterized by multiple glandular and fibrovascular structures within pleural tissue[2]. The epithelioid cells in the irregular small acinar lesions revealed positivity for TTF-1 and CK7, and negativity for CK20, CEA, and calretenin. The treatment of MRSA pleural space infection depends on its evolutive stage. The aim of this study was to establish the indications for videothoracoscopy in this rare pathology.

## CLINICAL SUMMARY

From January 2004 to December 2007, we observed 64 patients with pleural effusion due to MRSA pneumonia, already treated with linezolid injection for 2 ± 1 day. Patients were 41 males (64%) and 23 females (36%), with an average age of 42 ± 3 years (range: 28–69 years). All patients underwent computerized tomography (CT) of the thorax, fiberoptic bronchoscopy, primary intention drainage tube

32 Ch (under CT guidance in 12 patients), and pleural washing with specific antibiotic after microbiological examination of the pleural fluid. We used the linezolid as a local and systemic antibiotic in the following dosage: (1) 1200 mg in 500 ml of saline solution (0.9%) instilled in the pleural cavity two times per day by single application and (2) intravenous injection of 600 mg × 2/die administered routinely. This 3-day treatment was successful in 10 patients (16%) with a simple complicated parapneumonic effusion. No positive response was obtained in the other 54 (84%) who underwent video-assisted thoracoscopy (VAT). A complex complicated parapneumonic effusion was diagnosed in 22 patients (41%), and a simple and a complex empyema in 32 (59%), according to Light[3]. The VAT conversion to VATS (video-assisted thoracic surgery) allowed a complete lung parenchyma re-expansion in 22 multiloculated class 5 patients by means of breaking the septa and drainage-washing techniques continuously during 24 h. The postoperative average time of hospitalization was 3 ± 2 days. Thirty-two class 6 and 7 patients needed thoracotomy and diaphragmatic plastic with separated stitches due to the infection structure involvement (Fig. 1). In 20 of these (62%), we carried out thoracotomy after videothoracoscopic insufflation of carbon dioxide ($CO_2$) in order to separate coerced parenchyma and parietal pleural thickening (Fig. 2). This technique consisted of (1) the introduction of 8-mm trocar and 10-mm Veress-needle for the passage of an optical telescope (0° and 30°; Wolf Model) and $CO_2$ with a maximum flow of 9.9 l/min, respectively; (2) the achievement of the constant intrathoracic pressure between 12 and 18 mmHg and a volume of 3 or 4 l; (3) the easy thoracoscopic dissection of tissues, removing both the parietal and the visceral peel, started by means of endoscopic devices (Auto Suture endoshears 5 mm, endolung and endodissect 5 mm; Ethicon Endosurgery endoscopic blunt tip dissector) and completed via thoracotomy. The postoperative average time of hospitalization was 6 ± 1 day. In 12 of 32 patients (38%), this method was not used because of the reduction of cardiac output or the augmentation of pulmonary artery pressure. These patients showed prolonged postoperative air leaks (5 ± 2 days) because of the wide decortication via thoracotomy. Average time of hospitalization was 11 ± 1 day.

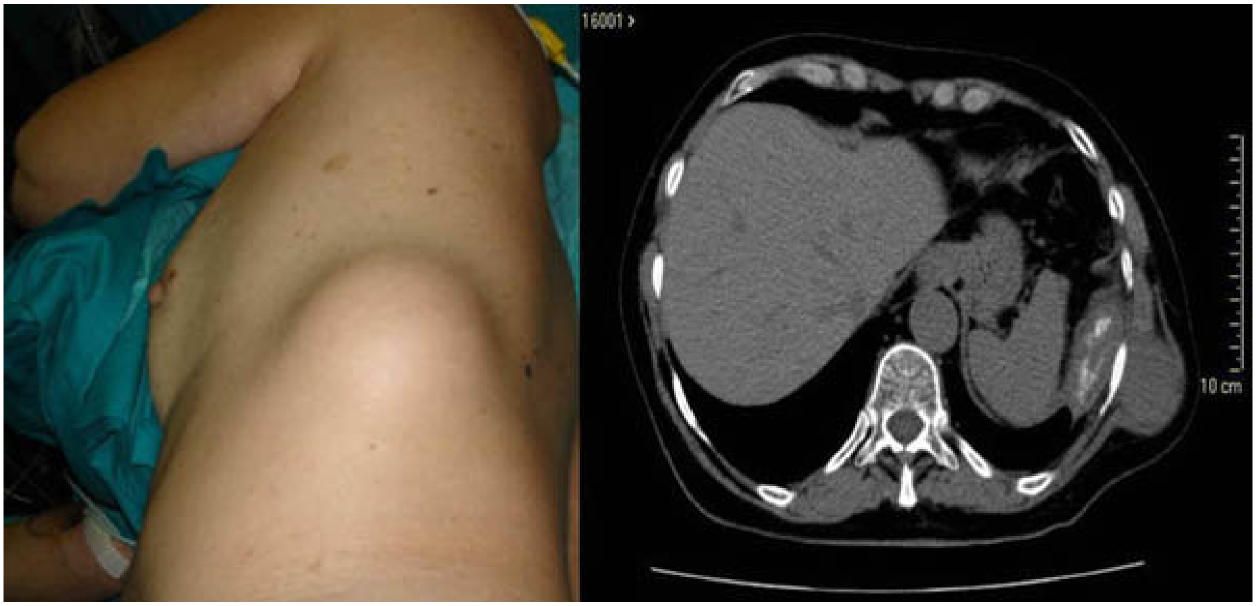

**FIGURE 1.** Empyema necessitatis. CT shows intrapleural, diaphragmatic, and chest localization due to a fistula in 8th intercostal space.

Clinical differences in the 64 patients are explained in Table 1.

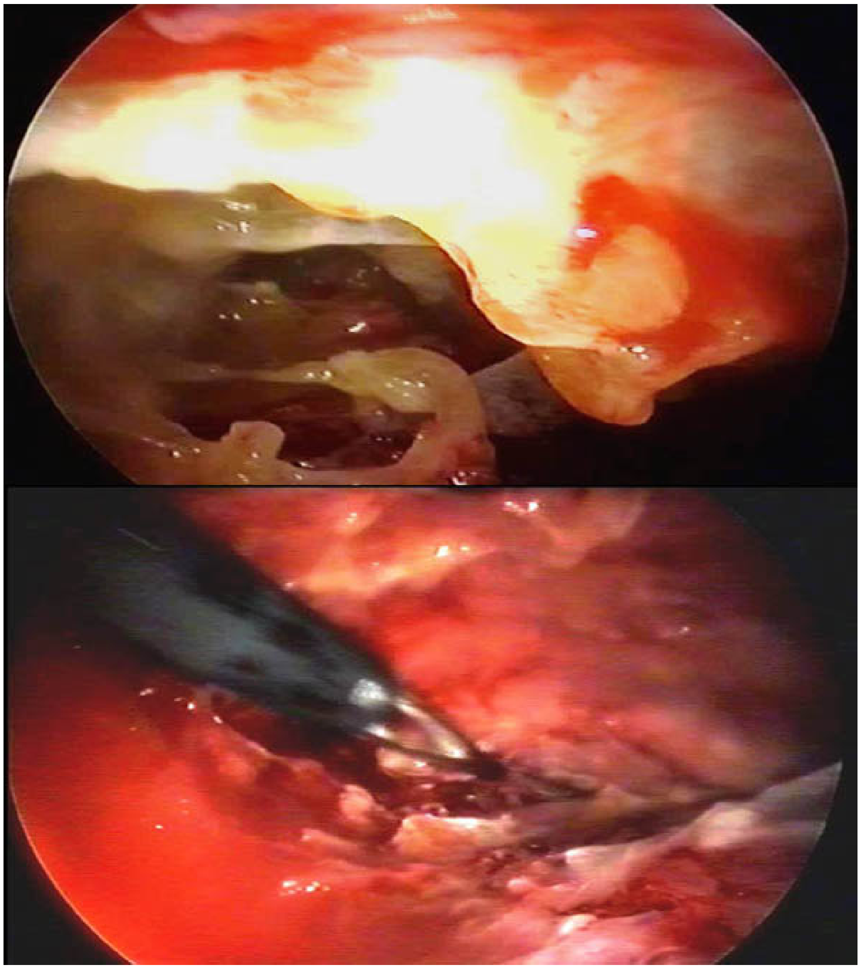

**FIGURE 2.** Pleural cavity loculations during videothoracoscopy $CO_2$ insufflation and dissection.

**TABLE 1**
**Clinical Status of Patients**

| Statement | Drianage Tube | VATS | VATS + THOR. | VAT + THOR. |
|---|---|---|---|---|
| Class 4 | 10 | — | — | — |
| Class 5 | — | 22 | — | — |
| Class 6–7 | — | — | 20 | 12 |
| Time of intervention | 5 ± 1 min | 21 ± 3 min | 90 ± 10 min | 120 ± 5 min |
| Time of drainage | 4 ± 1 day | 2 ± 1 day | 4 ± 1 day | 9 ± 1 day |
| Time of hospitalization | 6 ± 1 day | 3 ± 2 days | 6 ± 1 day | 11 ± 1 day |
| Number of patients | 10 | 22 | 20 | 12 |

VAT: Video-assisted thoracoscopy, VATS: video-assisted thoracic surgery, THOR.: thoracotomy.

## DISCUSSION

MRSA infection was characterized in our experience by rapid evolution to complex empyema and vast involvement of the diaphragm and chest wall. Treatment must proceed quickly to control sepsis, to evacuate pus, and to obliterate the pleural space, considering the high risk of mortality in this pathology[4]. VAT discloses the stage of empyema and factors responsible for unsuccessful tube thoracostomy therapy (84% in the personal study), orientating the surgical approach. Shankar et al.[5] revealed the resolution of infection by intercostal drainage under ultrasound guide rate of 92.3 and 62.5% in absence or in presence of septated empyema not correlated to MRSA disease. The use of thrombolytic enzymes pointed out by Lee et al.[6] and Lysy et al.[7] is almost obsolete and causes perplexity concerning (1) the dosage of fibrinolytic to be used, (2) the modes of administration, (3) duration of the treatment, (4) the stage of the disease in which fibrinolytic enzymes can be used, and (5) the low tolerability depending on the production of polypeptidergic substances. We opine that the extent of the infective-purulent process to the whole pleural cavity, with the development of multiple loculations, requires the direct breaking of adherences in videothoracoscopy and the positioning of a double drainage, respectively, at the apical and basal sites[8]. The aim of this procedure is to carry out continuous irrigations over 24 h, with subsequent moldering of the caseous material and again moving the lung parenchyma near the wall. The main advantages of videothoracoscopy are containment of surgical and anesthetic times, reduction of postoperative pain, rapid recovery of functions, reduced hospitalization, and the best esthetic results. Our experience, in accordance with the literature, states that the effectiveness of thoracoscopy strictly depends on the class of the empyema. Lackner et al.[9], in 17 patients at stage II, and Waller et al.[10], in 36 patients at stage II/III, achieved the drainage of the pleural cavity and decortication of the lung parenchyma by videothoracoscopy in 66 and 59%, with a conversion in thoracotomy rate of 24 and 41%. Cunniffe et al.[11], in 10 patients at stage II/III, and Cheng et al.[12], in 10 patients at stage II, realized videothoracoscopy decortication in 100% of patients and in 90% of patients without complications. We think that the empyemic process at class 6–7[3] does not permit reaching the pleural symphysis and the cavity obliteration with a mini-invasive procedure only, and for this very reason, open surgery is required. These considerations are more valid in MRSA pathology that displays a high tendency for local invasion. Basically, two conditions needed are (1) a metabolic-clinical status, allowing the invasive act that could cause bleeding and an atypical resection of parenchyma; and (2) the inactivity of pleural and pulmonary focuses. $CO_2$ insufflation during thoracoscopy is a new technique derived from laparoscopic pneumoperitoneum to facilitate the detection of a cleavage plan. This is an ideal method in complex fibrotic MRSA empyema and when applied before thoracotomy allows (1) better pleural and parenchymal exposition, (2) less tissue injury deriving from fibrosis, (3) lower incidence of wedge-resections, and (4) limited use of thoracoplasty for inadequate pulmonary re-expansion. The contraindications for such a technique: (1) absence of a small pleuropulmonary recess that inhibits the trocar introduction, (2) hemodynamic instability of the patient making increased intrapleural pressure impossible, and (3) presence of a wide pulmonary laceration ascribed to the primitive process with an augmentation of the risk of a gas embolism. In conclusion, MRSA empyema needs a rapid and aggressive approach due to failed therapy with drainage tube. We underline that VATS is the main treatment in class 5 of empyema. In class 6–7, videothoracoscopy $CO_2$ insufflation prompts an easier dissection, reducing time of open intervention and postoperative air leaks (Fig. 3).

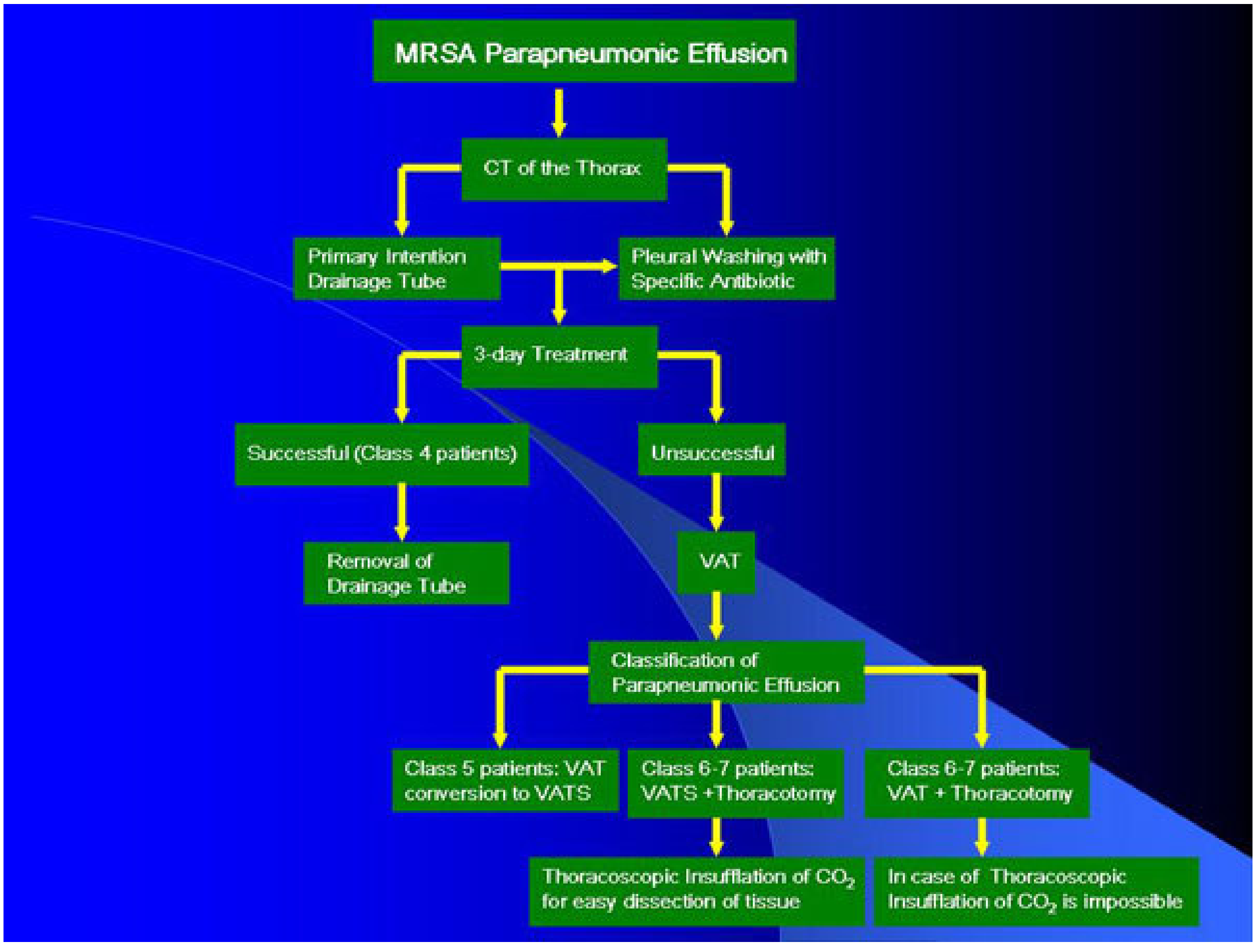

**FIGURE 3.** The flowchart of MRSA pleural effusion therapeutic approaches, according to our experience.
